# Cumulative weather effects can impact across the whole life cycle

**DOI:** 10.1111/gcb.14742

**Published:** 2019-07-25

**Authors:** Bethan J. Hindle, Jill G. Pilkington, Josephine M. Pemberton, Dylan Z. Childs

**Affiliations:** ^1^ Department of Animal and Plant Sciences University of Sheffield Sheffield UK; ^2^ Department of Applied Sciences University of the West of England Bristol UK; ^3^ School of Biological Sciences, Institute of Evolutionary Biology University of Edinburgh Edinburgh UK

**Keywords:** climate, covariation, density dependence, environmental variation, functional linear model, North Atlantic Oscillation, structural equation model, survival

## Abstract

Predicting how species will be affected by future climatic change requires the underlying environmental drivers to be identified. As vital rates vary over the lifecycle, structured population models derived from statistical environment–demography relationships are often used to inform such predictions. Environmental drivers are typically identified independently for different vital rates and demographic classes. However, these rates often exhibit positive temporal covariance, suggesting that vital rates respond to common environmental drivers. Additionally, models often only incorporate average weather conditions during a single, a priori chosen time window (e.g. monthly means). Mismatches between these windows and the period when the vital rates are sensitive to variation in climate decrease the predictive performance of such approaches. We used a demographic structural equation model (SEM) to demonstrate that a single axis of environmental variation drives the majority of the (co)variation in survival, reproduction, and twinning across six age–sex classes in a Soay sheep population. This axis provides a simple target for the complex task of identifying the drivers of vital rate variation. We used functional linear models (FLMs) to determine the critical windows of three local climatic drivers, allowing the magnitude and direction of the climate effects to differ over time. Previously unidentified lagged climatic effects were detected in this well‐studied population. The FLMs had a better predictive performance than selecting a critical window a priori, but not than a large‐scale climate index. Positive covariance amongst vital rates and temporal variation in the effects of environmental drivers are common, suggesting our SEM–FLM approach is a widely applicable tool for exploring the joint responses of vital rates to environmental change.

## 1 INTRODUCTION

Rapid climate change has led to increased interest in the responses of species and ecosystems to environmental variation (Ehrlen & Morris, 2015; Jenouvrier, 2013; Paniw, Maag, Cozzi, Clutton‐Brock, & Ozgul, 2019; Wolkovich, Cook, McLauchlan, & Davies, 2014). Identifying the underlying environmental drivers of vital rates is crucial for predicting how species abundances and distributions will be affected by future climate change (Ehrlen & Morris, 2015; Grosbois et al., 2008). Identifying the relevant drivers is challenging, because there may be a large number of possible candidate variables (Grosbois et al., 2008). Moreover, time lags between environmental events and demographic responses can occur (Forchhammer, Stenseth, Post, & Langvatn, 1998; Maldonado‐Chaparro, Blumstein, Armitage, & Childs, 2018), with the effect of a single driver potentially varying in magnitude and direction over time (Albon et al., 2017; Kruuk, Osmond, & Cockburn, 2015; Paniw et al., 2019; Tenhumberg, Crone, Ramula, & Tyre, 2018). Such lags between a climatic event and the demographic response may be caused by indirect effects, mediated through interactions with other species (Brown, 2011), or carry‐over effects (Norris, 2005), where the environment affects individual condition, resulting in delayed consequences for demographic rates such as survival (Ogle et al., 2015). Given the short temporal and spatial extent of most demographic data sets (Salguero‐Gomez et al., 2016) the number of possible effects can easily exceed the degree of temporal or spatial replication (Ehrlen, Morris, Euler, & Dahlgren, 2016). Methods that make efficient use of available data are, therefore, necessary to identify putative drivers and the temporal windows over which they act, and to accurately estimate the magnitude of their effects (Dahlgren, 2010; Ferguson, Reichert, Fletcher, & Jager, 2017; Teller, Adler, Edwards, Hooker, & Ellner, 2016).

Within a population the influence of environmental drivers typically varies according to individual state variables, such as age and sex (Gaillard, Festa‐Bianchet, Yoccoz, Loison, & Toigo, 2000). This necessitates structured approaches to predict population responses to future change (e.g. Jenouvrier et al., 2012). Stochastic structured models consider the means and variances of vital rates. These rates often exhibit positive temporal correlations, with higher reproductive rates in years with high survival and/or growth (Jongejans, Kroon, Tuljapurkar, & Shea, 2010; Nur & Sydeman, 1999). Positive correlations among the vital rates of different age–sex classes are also common. For example, years of high juvenile survival occur simultaneously with high adult survival and years that favour female survival also favour males (Rotella, Link, Chambert, Stauffer, & Garrott, 2012; Saether & Bakke, 2000). These positive covariances suggest the influence of common environmental drivers, yet these processes are typically considered independent (e.g. Coulson et al., 2001; Pokallus & Pauli, 2015). Multilevel demographic structural equation models (SEMs) allow the joint response of disparate vital rates and/or different age–sex classes to environmental variation to be captured using a biologically meaningful model (Hindle et al., 2018). SEMs have been widely adopted in ecology, for example to model the joint responses of multiple species to environmental change (e.g. Ovaskainen, Abrego, Halme, & Dunson, 2016; Warton et al., 2015). Their use is rare in single species demographic studies (though see Evans, Holsinger, & Menges, 2010; Hindle et al., 2018). Demographic SEMs introduce latent variable(s) to capture the covariation amongst the vital rates. These can be conceived as axes of common environmental variation, each of which may be driven by a combination of biotic and abiotic variables. The variation in each axis may thus be decomposed into the effects of different drivers, providing a simpler target for the challenging task of determining the underlying drivers of temporal variation than treating each demographic process independently (Hindle et al., 2018).

**Figure 1:**
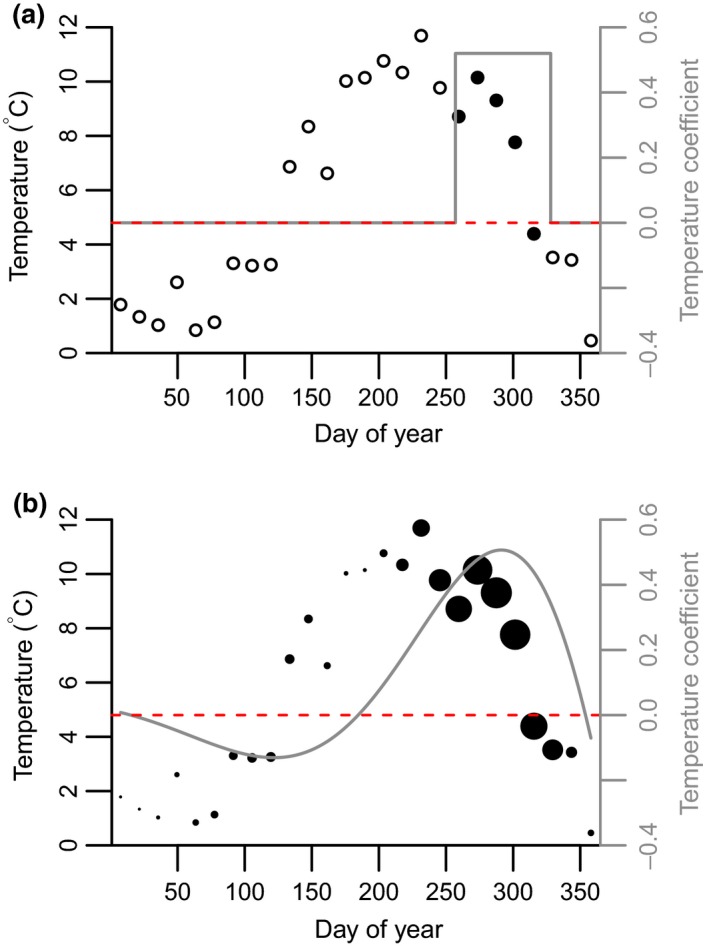
Schematic of (a) window‐based approaches and (b) functional linear model (FLM) approach to identifying climate effects. Points show means of raw temperature data calculated every fortnight over a single year. Grey lines show an example of the climate coefficients that could be generated under either type of approach. The red dashed line is at zero that is, where temperature has no effect; the effect of temperature is positive above this line and negative below it. Within each subplot the size of the points demonstrates their weight. Open points in (a) indicate where temperature is assumed to have no effect. In (a) the magnitude or direction of the temperature coefficients cannot differ within the chosen window (although multiple windows could be included), whereas in (b) both the magnitude and direction of the temperature coefficients can vary over the year. If the climate window is chosen a priori the position of the vertical grey lines in (a) is fixed, whereas under a sliding window approach the start and end of the window are estimated. The FLM can be estimated using spline basis expansion (see Equation 6)

When attempting to determine environmental drivers many studies consider a small number of putative drivers, each acting at a single time period (e.g. monthly means), chosen a priori based on expert knowledge of the focal species or closely related taxa (Figure 1a; Ogle et al., 2015; van de Pol et al., 2016). A mismatch between these time periods and the critical windows during which the vital rates are sensitive to variation in the environment will lead to poor predictions. Sliding window approaches, where an appropriate window is chosen by comparing the fit of models with different intervals, provide a partial solution to this problem (Figure 1a; van de Pol et al., 2016). However, a single window is usually selected (Husby et al., 2010; Stopher, Bento, Clutton‐Brock, Pemberton, & Kruuk, 2014; though see Kruuk et al., 2015), which does not allow the effect of a single variable to differ over time, despite evidence of this occurring in natural populations (Albon et al., 2017; Kruuk et al., 2015). Ecological responses to environmental factors are likely to be more similar at adjacent time points (Sims, Elston, Larkham, Nussey, & Albon, 2007; Teller et al., 2016). For example, the effect of high precipitation in February is likely to be more similar to that of high precipitation in March than August. Functional linear models (FLMs) allow the effect of environmental variables to be estimated as smooth, additive functions over time (Figure 1b; Roberts, 2008; Teller et al., 2016). This provides a biologically realistic framework for estimating climatic effects, allowing them to differ in magnitude and direction over the year, whilst making fewer a priori choices on the temporal extent of the effects.

**Figure 2:**
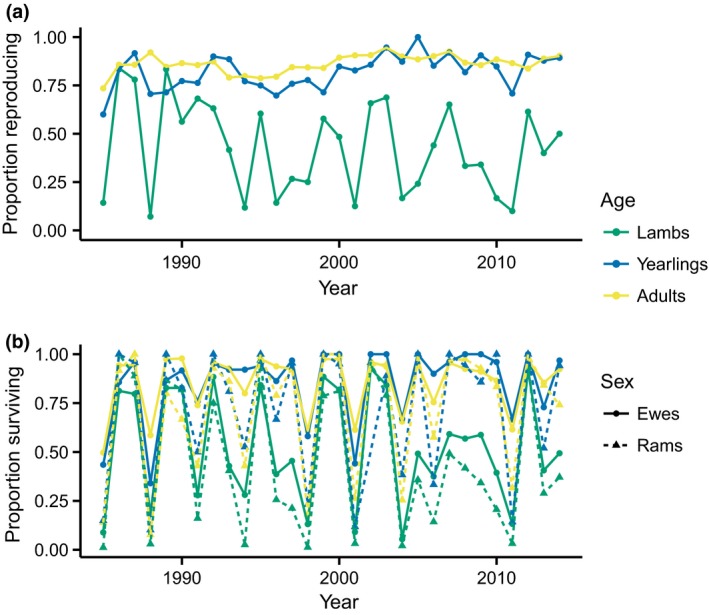
Observed proportion of individuals (a) reproducing (ewes only) and (b) surviving over the study period, separated by age–sex class

We investigated the dimensionality of the environment and decomposed the environmental variation into the effects of underlying drivers in a population of Soay sheep, *Ovis aries* (Clutton‐Brock & Pemberton, 2004). This population exhibits pronounced density‐dependent fluctuations, with high survival and fecundity at low densities and population crashes often occurring at high densities (Clutton‐Brock, Price, Albon, & Jewell, 1991, 1992). However, high densities do not always result in crashes, suggesting the population responds to an interaction between density and the abiotic environment (Clutton‐Brock & Pemberton, 2004; Coulson et al., 2001). Previous studies have found that harsh winter weather conditions, such as wet and windy weather, decrease survival and fecundity (Berryman & Lima, 2006; Catchpole, Morgan, Coulson, Freeman, & Albon, 2000; Coulson et al., 2001, 2008; Grenfell et al., 1998; Milner, Elston, & Albon, 1999; Stenseth et al., 2004). These studies have typically either used a large‐scale index (winter North Atlantic Oscillation [NAO]; e.g. Berryman & Lima, 2006; Stenseth et al., 2004) or have chosen the temporal windows of putative local drivers a priori (Catchpole et al., 2000; Coulson et al., 2001), focusing on the winter period, when the vast majority of mortality occurs (Hallett et al., 2004). Longer term climatic effects have not been considered. Moreover, there are strong temporal correlations among the different vital rates, across sex and age classes, with years of high lamb, yearling, and adult survival occurring simultaneously with years of high reproduction (Figure 2; Coulson, Albon, Pilkington, & Clutton‐Brock, 1999). We used a demographic SEM to show that the temporal component of the variation in demographic rates is relatively low dimensional—just two axes of environmental variation are required to explain the temporal variation in survival, reproduction and twinning across six age–sex classes. We then decomposed the first axis of environmental variation into the effects of density, a temporal trend, and climatic covariates, using FLMs to determine the critical window over which three local weather variables and NAO acted. We compared the predictive performance of the FLMs both to using a large‐scale climate index and to selecting the critical window for a local weather variable a priori.

## 2 STUDY SYSTEM

We used 30 years of demographic data (1985–2014) on a population of Soay sheep in the Village Bay area of Hirta, in the St Kilda archipelago off the North‐West of Scotland (Clutton‐Brock & Pemberton, 2004). Nearly 100% of newborn lambs are tagged within days of birth. Population censuses are carried out three times a year and mortality searches ensure the fate of most individuals is known. The lack of large herbivorous competitors and predators of the adults mean the population dynamics are largely driven by intraspecific competition for food (Clutton‐Brock & Pemberton, 2004).

For the FLMs, we considered NAO and three local weather variables; minimum temperature, precipitation, and maximum wind speed. Cold, wet, and windy conditions may increase heat loss (McArthur & Ousey, 1996; Webb & King, 1984; Webster & Park, 1967) and reduce grazing due to increased time spent sheltering (Stevenson, 1994). Cold, wet weather may also have indirect effects through spring–summer vegetation growth and subsequent food availability. The predictive performance of the FLMs was compared to two reference models; using a large‐scale climate index (December–March NAO, referred to herein as winter NAO; Coulson et al., 2001; Stenseth et al., 2004) and a local weather variable with the critical window selected a priori (March precipitation; Catchpole et al., 2000; Coulson et al., 2001). High winter NAO values are associated with warm, wet, and windy weather in northern Europe (Hurrell & VanLoon, 1997) and decreased survival and fecundity in this population (Coulson et al., 2001; Stenseth et al., 2004). Between January and March the body weight of the sheep can decline by as much as 30% (Clutton‐Brock et al., 1997); high precipitation at the end of this period, before the onset of rapid new vegetation growth, thus appears likely to decrease survival. The winter NAO model differs from the NAO FLM, where monthly NAO values were included over a 19 month period.

NAO data were obtained from the National Center for Atmospheric Research (https://climatedataguide.ucar.edu/climate-data; Hurrell, 1995). Daily local weather data were acquired from Stornoway meteorological office, the closest weather station open for the entire study period (approximately 140 km from St Kilda; data available from badc.nerc.ac.uk). These data were closely correlated with those from St Kilda from 1999 onwards (when weather stations were set up on site; temperature, *r* = .97, precipitation, *r* = .85, wind speed, *r* = .93; Figure S1). Missing data (<1% of temperature and precipitation and 6% of wind data) were interpolated using the *forecast* package (Hyndman & Khandakar, 2008) in R (R Core Team, 2016).

## 3 STRUCTURE OF THE SEM

Demographic SEMs (see Hindle et al., 2018 for more detail) excluding climatic drivers were constructed to explore the number of axes required to account for the temporal covariation among the vital rates and to provide a baseline to evaluate the predictive performance of the climatic models. The population was split into three age classes: lambs (0–1 year), yearlings (1–2 years), and adults (>2 years). Female reproduction is not limited by male availability. A small proportion of yearling and adult ewes produce twins each year (Clutton‐Brock & Pemberton, 2004). The demographic SEMs, therefore, included 11 submodels: August (*t*) to August (*t* + 1) survival of each age–sex class (six submodels, *s* superscript), spring reproduction of ewes in each age class (three sub‐models, *r* superscript), and twinning of yearling and adult ewes (two sub‐models, *t* superscript).

**Figure 3:**
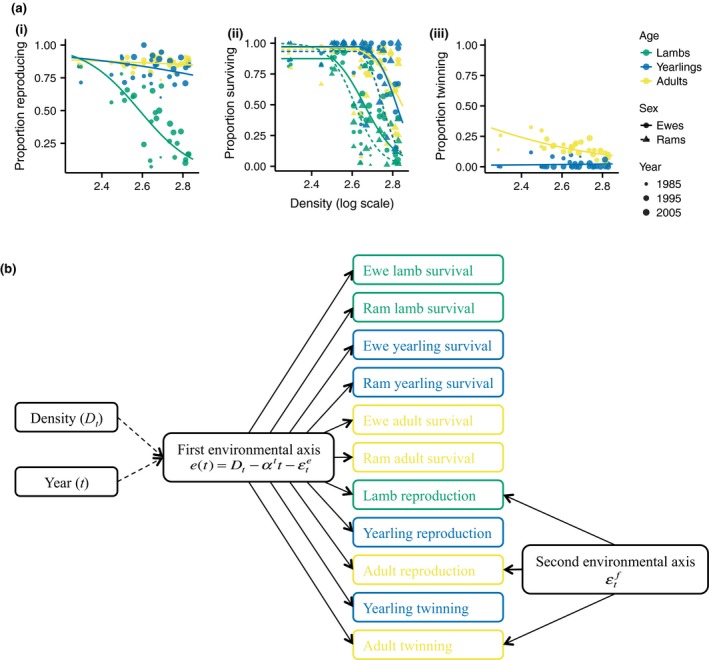
(a) Proportion of individuals (i) reproducing, (ii) surviving, and (iii) twinning against population density, separated by age–sex class. Points show observed data. Point size is on a continuous scale, with larger points indicating later years in the study. Lines show fitted baseline submodels (Equation 3) for the two‐axis model for the midyear of the study, with the random year effect at zero, using the posterior medians. (b) Path diagram for the two‐axis model. Colours denote the age class and match those used in (a). The vital rates are given by Equations ((1), (2), (3), (4)). Note that the structure of the single‐axis model is the same, excluding the second environmental axis

We initially fitted a highly constrained model that assumes temporal (co)variation in the vital rates is driven by a single time‐varying environmental axis (*e*) common to all 11 submodels (the single‐axis model). When resource availability is high (low sheep densities) differences in the environment have little effect on survival (Figure 3a; Grenfell et al., 1998). The probability of survival (*S*) in year *t* for each age–sex class (except ram lambs—see below) was, therefore, estimated using threshold models (Figure 3a), assuming a binomial distribution:

(1) $$\text{logit}\;\left(S_{∙,t}\right)=\left\{\begin{array}{cc} \beta_{∙}^{0,s}+\beta_{∙}^{t,s}t & \text{if}\;e\left(t\right)<\theta_{∙},\\ \beta_{∙}^{0,s}+\beta_{∙}^{t,s}t-\beta_{∙}^{e,s}\;\left(e\left(t\right)-\theta_{∙}\right) & \text{if}\;e\left(t\right)⩾\theta_{∙}, \end{array}\right.$$

where *β* ^0^ are intercepts, *β^t^* and *β^e^* are slope terms for a temporal trend and the first environmental axis (*e*) respectively, and *θ* are thresholds. The • subscript indicates parameters estimated separately for each age–sex class. There was no evidence of a threshold in the fecundity (reproduction or twinning) or ram lamb survival submodels (Figure S2). The probability of reproduction (*R*) was estimated using a simple logistic regression:

(2) $$\text{logit}\;\left(R_{∙,t}\right)=\beta_{∙}^{0,r}+\beta_{∙}^{t,r}t-\beta_{∙}^{e,r}e\left(t\right),$$

with the parameters defined as above (Equation 1). The twinning and ram lamb survival submodels were structurally analogous to Equation (2).

As the vital rates are highly density dependent and population sizes have generally increased over the study period (Figure S3; Coulson et al., 2008) the environmental axis (*e*) was modelled as a function of density (*D_t_*; the log 10 number of sheep in the population in August of year *t*) and the study year (*t*):

(3) $$e\left(t\right)=D_{t}-\alpha^{t}t-\varepsilon_{t}^{e},$$

where *α^t^* is a slope term for the temporal trend. The random year effects ($\varepsilon_{t}^{e}$) account for residual covariation among the vital rates and were sampled from a normal distribution with mean zero and *SD* *σ^e^*. Including a temporal trend (*α^t^t*) here allows for an interaction between density and time across the vital rates, whilst the vital rate‐specific temporal trends (given by *β^t^t* in Equations 1 and 2) allow for temporal trends in the mean vital rates (Figure S4).

We used a Bayesian framework for inference. Parameter estimates were obtained using Markov Chain Monte Carlo (MCMC) simulation in JAGS (Plummer, 2003), using the R package *runjags* (Denwood, 2016). Weakly informative priors were used to aid convergence (Table S1). The models were run using two chains, each with a discarded burn‐in period of 1 × 10^5^ iterations. The chains were run for a further 6 × 10^6^ iterations, and thinned, keeping every 2,000th sample to produce a total posterior sample of 6,000 across both chains. Posterior predictive checks were used to determine whether the temporal variation in the vital rates was well explained by the initial model (Gelman, Carlin, Stern, & Rubin, 2004).

## 4 DEVELOPMENT OF THE SEM

**Figure 4:**
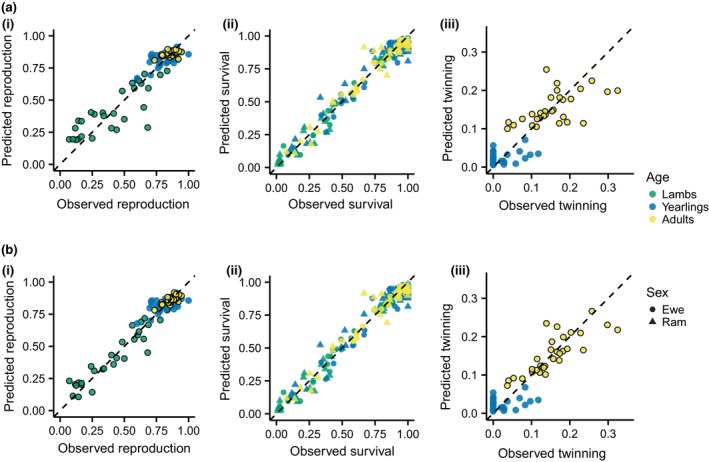
Observed and predicted vital rates using (a) the single‐axis and (b) two‐axis model. Black borders around the points indicate those processes partially driven by the second axis (Figure 3b). The addition of the second axis increases the correlation between observed and predicted vital rates from .47 to .74 for adult reproduction, .58 to .81 for adult twinning, and .84 to .94 for lamb reproduction. The vital rates driven by the primary environmental axis only (e.g. survival for all demographic classes) were not affected by the addition of the second axis. Vital rates were predicted using the posterior medians as the parameter estimates, the observed density from *t* − 1 and including the estimated random effect for each year. Dashed lines show a 1:1 correlation

Survival across the six age–sex classes was well predicted by the single‐axis model (Figure 4a). However, posterior predictive checks revealed evidence of unexplained variation in the fecundity submodels (Figure 4a; Appendix S1). Independent, submodel specific random year effects were introduced into the fecundity submodels to explore this unexplained variation (Appendix S1). The posterior distributions of the corresponding variance terms were concentrated at zero for the yearling reproduction and twinning submodels. However, the variances of the remaining fecundity components were nonzero, and the associated year effects were positively correlated (Appendix S1). Consequently, we constructed a two‐axis model (Figure 3b) by introducing a second latent variable affecting lamb reproduction, adult reproduction, and adult twinning only. The probability of lamb or adult reproduction was then given by:

(4) $$\text{logit}\;\left(R_{∙,t}\right)=\beta_{∙}^{0,r}+\beta_{∙}^{t,r}t-\beta_{∙}^{e,r}e\left(t\right)+\beta_{∙}^{f,r}\varepsilon_{t}^{f},$$

where *β^f^* is the slope for the second environmental axis, $\varepsilon_{t}^{f}$. The adult twinning submodel is structurally analogous to Equation (4). The random year effects for the first and second environmental axes ($\varepsilon_{t}^{e}$ [Equation 3] and $\varepsilon_{t}^{f}$ respectively) were allowed to covary. Thus, these were sampled from a multivariate normal distribution with means of zero and covariance matrix Σ.

The vital rates were well predicted using the two‐axis model (Figure 4b). Adding the second environmental axis improved the fit of the lamb reproduction, adult reproduction, and adult twinning submodels (Figure 4). However, it was the first axis that captured most of the variation in the vital rates across the lifecycle. The 95% credible intervals of the *β^e^* slope terms overlap zero in only two of 11 submodels (adult reproduction and yearling twinning; Appendix S1), indicating that the first axis drove variation in survival and fecundity in nearly all age–sex classes. Variation in survival across the age–sex classes and the majority of variation in the most variable fecundity rate (lamb reproduction) was captured by the first axis (Figure 4). Variation in yearling twinning was not captured by either axis, however, this remains low throughout the study period and is likely to be explained by demographic stochasticity (the maximum number of yearlings twinning in 1 year was three and none of the yearlings twinned in 19 out of the 30 years). There was no evidence of correlations between the yearly estimates of the second environmental axis ($\varepsilon_{t}^{f}$) and density (*D_t_*) or year (*t*; Appendix S1), indicating these effects were captured by the first axis. There was also no evidence of a correlation with the sex ratio (Appendix S1), suggesting female fecundity was not limited by male availability.

## 5 IDENTIFYING CLIMATIC DRIVERS

We used the two‐axis model for further analysis of environmental effects. Here, we consider the first environmental axis (*e*), which drives the majority of the covariation in the vital rates (Figure 4). We found no evidence of weather conditions driving the variation in the second axis (Appendix S2.2). In the reference models, the first environmental axis was given by:

(5) $$e\left(t\right)=D_{t}-\beta^{m}M_{t}-\alpha^{t}t-\varepsilon_{t}^{e},$$

where *M_t_* is the climatic variable (winter NAO or mean March precipitation) in year *t* and *β^m^* is a slope term. For the local weather FLMs the means of the daily variables every fortnight (*w*) from the beginning of January in *t* − 1 (*w* = 1) until the end of July in *t* (*w* = 42), were used as covariates (Figure S5). Monthly NAO data over the same time period were used for the NAO FLM (*w = *1,2 …, 19). Seasonality was removed from the weather data by centering (Figure S5). As the seasonal component of the climatic signal does not vary among years it cannot explain the interannual variation in the demographic rates, however, removing the seasonality from the data can aid convergence of the models. Each covariate was included in a separate model, with the first environmental axis (*e*) given by:

(6) $$e(t)=D_{t}-\sum_{w=1}^{W}f_{c}\left(w\right)C_{\mathrm{tw}}-\alpha^{t}t-\varepsilon_{t}^{e},$$

where *C_tw_* is climate variable *C* in year *t* and time interval *w* (fortnight for the local variables and month for NAO) and *f_c_*(w) is a smooth function that allows the effect of the climate covariates to vary smoothly over the 19 month period (e.g. Figure 1b). The FLM was estimated using eight knots and a cubic regression (“cr”) spline basis (see Appendix S2.1). Teller et al. (2016) and Tenhumberg et al. (2018) provide detail on using FLMs to estimate demographic rates using lagged climatic data (see Wood, 2017 for more general detail). Example code for using the SEM‐FLM approach is available on github (https://doi.org/10.5281/zenodo.3236766).

The out of sample predictive performance and the proportion of variance (*R* ^2^) in *e* explained for each of the FLMs (Equation 6) was compared to the base model (Equation 3), and the reference models (Equation 5). Leave one out cluster cross validation was used to assess predictive performance. The models were refitted 30 times, leaving out each year of data in turn. The predictive performance of each model was estimated using the expected logwise predictive density ($\hat{\text{elpd}}$; Vehtari, Gelman, & Gabry, 2017). Since ignoring the random year effects (*ε^e^* and *ε^f^*) may lead to overly optimistic estimates of a model's predictive performance (Pavlou, Ambler, Seaman, & Omar, 2015; Skrondal & Rabe‐Hesketh, 2009), a Monte Carlo approach was used to calculate the marginal predictive density. The $\hat{\text{elpd}}$ was then

(7) $$\hat{\text{elpd}}=\sum_{i=1}^{n}\log\left(\frac{1}{\text{SM}}\sum_{s=1}^{S}\sum_{m=1}^{M}p\left(y_{i}|\theta^{s,m}\right)\right),$$

where *S* is the number of draws from the posterior, *M* is the number of samples from the random year effect distributions and *n* is the number of years of data (Vehtari et al., 2017). The likelihood *p*(*y_i_*|θ*^s,m^*) is calculated as the product of the likelihoods for each of the 11 submodels; *y_i_* is the observed data in year *i* and *θ^s,m^* is draw *s* from the posterior of the model that excluded the data from year *i*, with sample *m* from the random effects. Posterior samples were obtained using MCMC sampling in JAGS as above and the $\hat{\text{elpd}}$ was estimated using the whole posterior sample of 6,000 for each year. *ε^e^* and *ε^f^* were sampled from a multivariate normal distribution 1,000 times for each posterior sample. The difference in the predictive ability of two models (A and B) on the deviance scale was given by $-2\;(\hat{elpd^{A}}-\hat{elpd^{B}})$ (Vehtari et al., 2017).

## 6 CLIMATIC MODEL RESULTS

**Figure 5:**
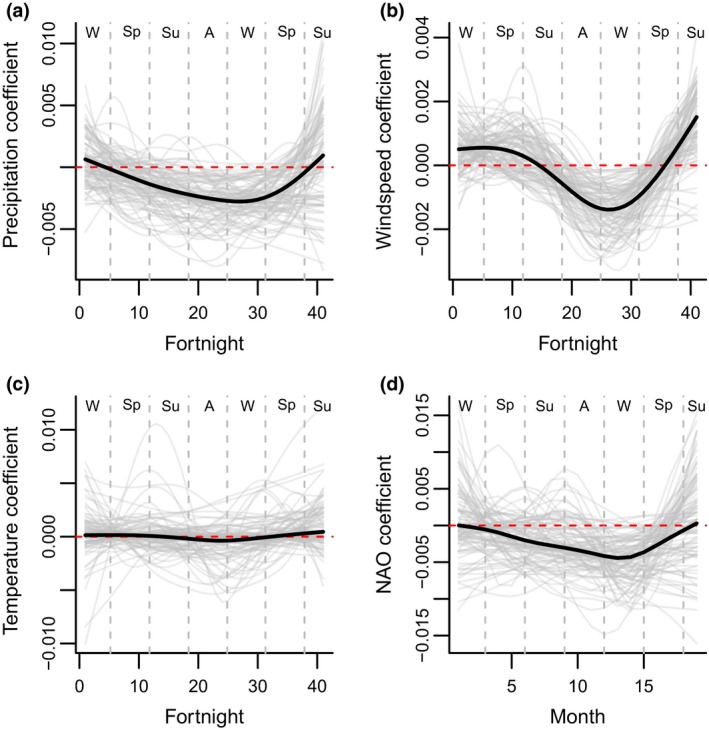
Functional linear models for (a) precipitation, (b) wind speed, (c) temperature, and (d) North Atlantic Oscillation (NAO). Thick black lines show the posterior medians, thinner grey lines show 100 simulations from the posterior. The horizontal dashed red line is at 0. Dashed vertical lines and letters at the top of the plot indicate the seasons. Coefficients above the line indicate that higher values of the weather variable during that time period were associated with an increase in survival and fecundity. The rut occurs during autumn and mortality occurs during winter and early spring

The strongest weather effects were over winter, when the vast majority of mortality occurs (Hallett et al., 2004), but there was also evidence of longer term effects, especially during autumn (Figure 5). The vital rates were driven by the cumulative effect of precipitation from summer *t * − 1 until winter in year *t*. Over this time period, increased precipitation decreased survival and fecundity, with the strongest effects in autumn and winter (Figure 5a). High wind speeds had a positive effect in winter and spring *t* − 1 and a negative effect over autumn and winter in *t* (Figure 5b). Higher NAO values from spring in *t * − 1 were associated with decreased survival and fecundity, with particularly strong effects over winter in year *t* (Figure 5d).

Cross validation was not carried out on the temperature FLM, as there was no evidence of an effect on the vital rates (Figure 5c). All of the remaining climatic models had a better predictive performance than the baseline model (Table 1), however, the gain was marginal in the case of March precipitation. Wind speed was the best performing of the FLMs; wind speed and precipitation both outperformed the monthly NAO FLM (Table 1). However, the winter NAO model had a better predictive performance than any of the FLMs, with higher winter NAO values associated with decreased survival and fecundity (Table 1). Using an additive framework to include both precipitation and wind speed in a single model did not improve the predictive performance beyond the wind speed FLM (Appendix S2.3). Models including the precipitation or wind speed FLM as well as winter NAO exhibited marginally better predictive performance than the winter NAO model (Appendix S2.4).

**Table 1:** Performance of the climatic models

Model	Relative predictive performance	*R* ^2^
March precipitation	−0.67	.68 (.58–.74)
Monthly NAO FLM	−1.96	.77 (.63–.84)
Fortnightly precipitation FLM	−3.82	.77 (.65–.84)
Fortnightly wind speed FLM	−5.73	.81 (.69–.87)
Winter NAO	−13.57	.86 (.79–.90)

Note

Relative predictive performance is the difference in out of sample predictive performance (Equation 7) between the baseline model (with no climate effects; Equation 3) and each climate model, on the deviance scale. More negative values indicate models with a better predictive performance. *R* ^2^ is the proportion of variation in the first environmental axis (*e*) explained by the fixed effects (i.e. density, the temporal trend, and the relevant climatic variables). Values are the median and 95% quantiles, calculated by sampling from the posterior distribution. *R* ^2^ for the base model is .68 (.57–.74).

Abbreviation: FLM, functional linear model; NAO, North Atlantic Oscillation.

## 7 DISCUSSION

Our analysis shows that vital rates can vary along considerably fewer temporal dimensions than the number of vital rate–age–sex combinations to be estimated. Using a demographic SEM to simultaneously estimate the vital rates provides a simple target for the challenging task of decomposing temporal variation in the vital rates into the effects of different intrinsic (e.g. density) and extrinsic (e.g. climatic) covariates. In the Soay population a simple demographic SEM with a single axis described most of the variation in survival, reproduction, and twinning across six age–sex classes, with all vital rates well‐predicted by two environmental axes. Age and sex differences in the mean and variance of vital rates (Gaillard, Festa‐Bianchet, & Yoccoz, 1998; Gaillard et al., 2000) have led to vital rates for different age–sex classes being treated independently (e.g. Coulson et al., 2001). Positive covariances amongst the vital rates across age–sex classes suggest that, despite differences in the magnitude of environmental effects, common factors drive the variation in vital rates across age–sex classes. Such positive covariances are widespread in natural populations, including in plants (Jongejans et al., 2010), birds (Nur & Sydeman, 1999), and mammals (Rotella et al., 2012), suggesting the SEM approach is widely applicable.

Despite many previous attempts to determine underlying drivers in the Soay sheep population, (e.g. Catchpole et al., 2000; Clutton‐Brock et al., 1991, 1992; Coulson et al., 2001; Grenfell et al., 1998; Milner et al., 1999; Stenseth et al., 2004), we identified previously unseen weather effects using the FLMs, with the vital rates affected by cumulative effects from up to 12 months prior to the mortality period. The choice of possible drivers, and the periods over which they are assumed to act, are important modelling decisions, yet many studies provide little justification for their chosen variables (Ehrlen et al., 2016; van de Pol et al., 2016). Our analysis reinforces previous results, whereby increased wind speeds and precipitation over winter increase mortality (Coulson et al., 2001; Milner et al., 1999). However, we also found that high precipitation and wind speeds during the autumn rut appeared nearly as costly as during winter, demonstrating that the FLM method can uncover novel climatic effects even in well‐studied populations. Rutting is energetically costly, with decreased foraging time and increased energy expenditure (Stevenson & Bancroft, 1995). Environmental conditions during this period may, therefore, have substantial effects on body condition and subsequent survival (Barboza, Hartbauer, Hauer, & Blake, 2004).

The weight of individuals in summer is not associated with population density in the previous winter (Clutton‐Brock et al., 1991), indicating that by summer individuals are able to regain their condition following harsh winters. Thus it appears unlikely that winter conditions will create interannual carry‐over effects. However, we found that higher maximum wind speeds in winter and spring *t* − 1 appeared to increase survival in year *t*. Sequential density dependence, where harsh environmental conditions decrease density resulting in higher survival (Rakhimberdiev, Hout, Brugge, Spaans, & Piersma, 2015), could lead to delayed climatic effects. However, the inclusion of density during the summer (i.e. after the higher wind speeds effect) in this case suggests the lagged effect is independent of any density effects. The mechanisms by which this lagged effect of wind speed may be operating thus need further research. It is possible that wind speed is merely correlated with another environmental driver (Ehrlen et al., 2016; Grosbois et al., 2008) that underpins an indirect, delayed effect of the vegetation (Terraube et al., 2015). However, the lack of evidence of lagged effects of temperature, which would be likely to drive changes in vegetation productivity (Hunter & Grant, 1971), is suggestive of a lack of indirect effects via resource availability in this population.

The relative importance of precipitation and windspeed, compared to temperature, explains why NAO is a good predictor of the vital rates in this population as higher winter NAO values are associated with wetter and windier, yet warmer, winters in the study area (Hurrell & VanLoon, 1997). Predictions of future NAO are widely variable, depending on the model and emissions scenario used, however, overall the pattern suggests that the mean NAO will increase by the end of the century (Simmonds & Coulson, 2015). Given the negative relationship between winter NAO and the demographic rates, found in this study and many previous studies (e.g. Coulson et al., 2001; Stenseth et al., 2004), population sizes may be likely to decrease in the future (Simmonds & Coulson, 2015). This is supported by predictions in the local climatic variables, where increases in winter precipitation and wind speeds, along with decreases in summer wind speeds across the United Kingdom (Murphy et al., 2019) would also be predicted to lead to a decrease in population size.

We have demonstrated that the use of flexible statistical tools to determine the temporal windows over which local variables act can improve their predictive performance relative to a priori decisions about the relevant windows. However, all three local variables were still outperformed by a large‐scale climatic index (winter NAO). Such large‐scale indices have often been used as a proxy for, and have frequently outperformed, local weather variables (Hallett et al., 2004; Post & Stenseth, 1999). Despite this, the relationship between large‐scale indices and local weather may be temporally and/or spatially variable (Anders & Post, 2006; Stenseth et al., 2003). Thus large‐scale indices may provide inaccurate future predictions of population dynamics, whilst using such indices to compare the sensitivity of populations to climatic conditions across large spatial scales may simply recover patterns in the strength of the relationship between the index and local weather variables (Anders & Post, 2006; van de Pol et al., 2013). A likely reason for the relatively high predictive performance of large‐scale indices is that they incorporate the effects of multiple local variables. Although interactions between local variables are likely to be important (Ehrlen et al., 2016; Stenseth & Mysterud, 2005), including multiple climatic drivers is not simple, as they are often correlated (Grosbois et al., 2008). The choice of local weather variables versus large‐scale indices for such studies depends, therefore, on the study system and spatial and temporal extent of the study as well as its purpose. Large‐scale indices do not directly influence vital rates, which means their use cannot improve the mechanistic understanding of how populations respond to environmental variation (Stenseth et al., 2003) without also considering the associations between such indices and local weather conditions (Almaraz & Amat, 2004; Anders & Post, 2006). If the aim of the study is to predict dynamics under future change the variable with the highest predictive performance, which frequently is a broadscale index, is probably most appropriate. Alternatively, if the aim is to understand how a population responds to environmental variation local climatic drivers may be more relevant (Nielsen et al., 2012).

The 30 year data set used in this analysis has higher levels of temporal replication than the majority of demographic data sets (Salguero‐Gomez et al., 2016, 2015). The SEM component of the model can work well with more limited temporal replication, for example, Hindle et al. (2018) used a 8 year data set. The FLM part of the approach has higher data requirements, however, with Teller et al. (2016) suggesting that 20–25 years of data may be needed to accurately identify environmental drivers and precisely estimate their effects. However, alternative approaches also perform relatively poorly with low temporal replication (van de Pol et al., 2016), suggesting this issue is due to the complexity of the problem rather than being specific to FLMs. As with any statistical approach checking the fit of the model is important and can help to improve predictive performance. Cross validation is rarely used in studies of the environmental drivers of demographic rates (Grosbois et al., 2008). When identifying environmental drivers the aim is often to predict population responses to a future change in those drivers (Gotelli & Ellison, 2006); thus, out‐of‐sample predictive performance is a key measure of model utility (Wenger & Olden, 2012). Many studies instead rely on within‐sample measures, such as Akaike information criteria (AIC), which may be subjected to overfitting (Dahlgren, 2010; Murtaugh, 2009; Raffalovich, Deane, Armstrong, & Tsao, 2008; van de Pol et al., 2016). Moreover, the use of measures such as AIC is not straightforward for hierarchical models, which are typically necessary for dependent demographic data clustered in time or space (Vaida & Blanchard, 2005).

Rapid climate change has increased interest in predicting ecological responses to environmental variation. For accurate predictions relevant drivers and their temporal windows of influence must be identified and their effects must be accurately quantified. We have demonstrated that the dimensionality of the environment can be remarkably low, suggesting the influence of common environmental drivers across the vital rates and life cycle, and thus providing a simpler target for identifying such drivers. By incorporating climatic drivers over extended temporal periods FLMs can increase the predictive performance of local variables. Including interactions among climatic variables may further increase the predictive performance of local models, beyond that of large‐scale indices (Stenseth & Mysterud, 2005).
