# Supplementary material for: Cumulative weather effects can impact across the whole life cycle
Source: Glob Chang Biol. 2019 Jul 25;25(10):3282–93. doi: 10.1111/gcb.14742 (PMC6771737; doi:10.1111/gcb.14742)
Supplement: Supplementary file 1 [file GCB-25-3282-s001.pdf]

Supplementary figures

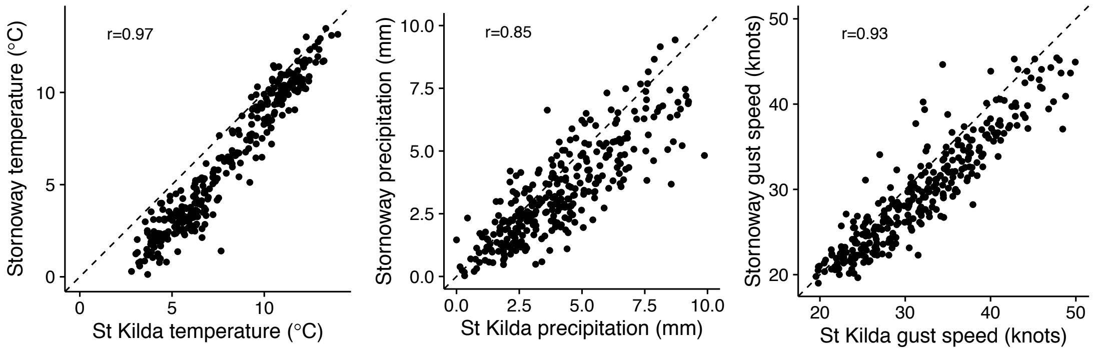

Figure S1: Correlation between minimum temperature, mean precipitation and maximum windspeed on St Kilda and from the meteorological office station at Stornoway airport (150km away). Dashed lines show 1:1 lines.

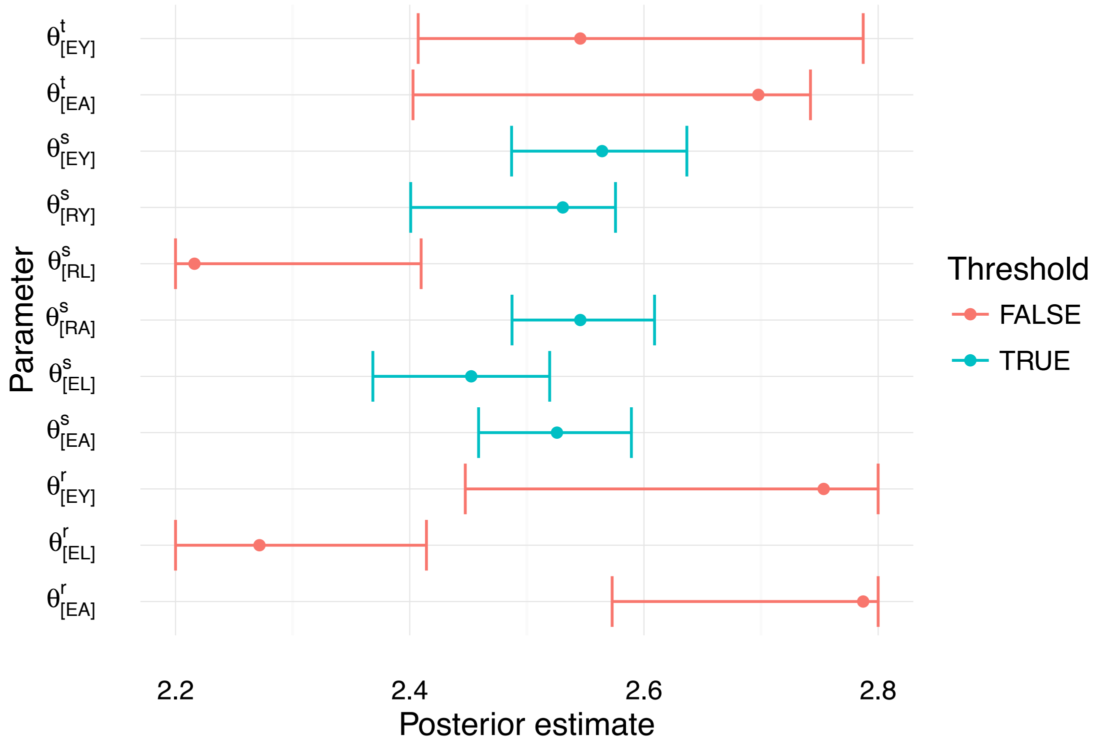

Figure S2: Posterior distributions (mode and 95% credible intervals) for model using threshold models for all of the vital rate sub-models (see equation 1 in main text). Subscripts show the sex and stage class (i.e. EA is ewe adult) and superscripts show the vital rate (survival, reproduction or twinning). Colours denote whether or not the threshold parameter is retained in that sub-model. The posteriors of the threshold parameters for the survival sub-models (with the exception of ram lamb survival) are well defined, whilst those of the fecundity models and ram lamb survival are generally much broader and often focused at either end of the prior (see Qian 2014).

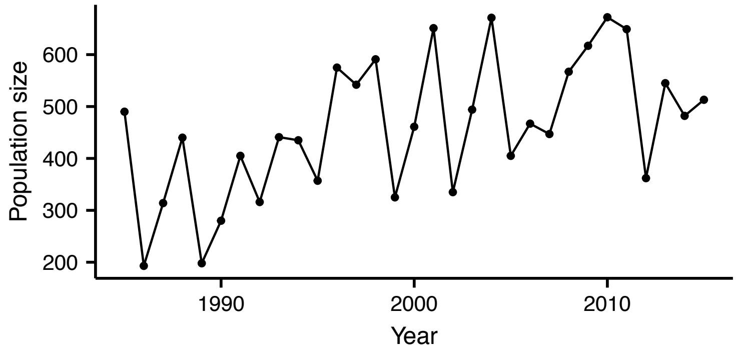

Figure S3: Total population size in the Village Bay area of Hirta over the study period. Whilst highly variable the number of individuals has generally increased over time.

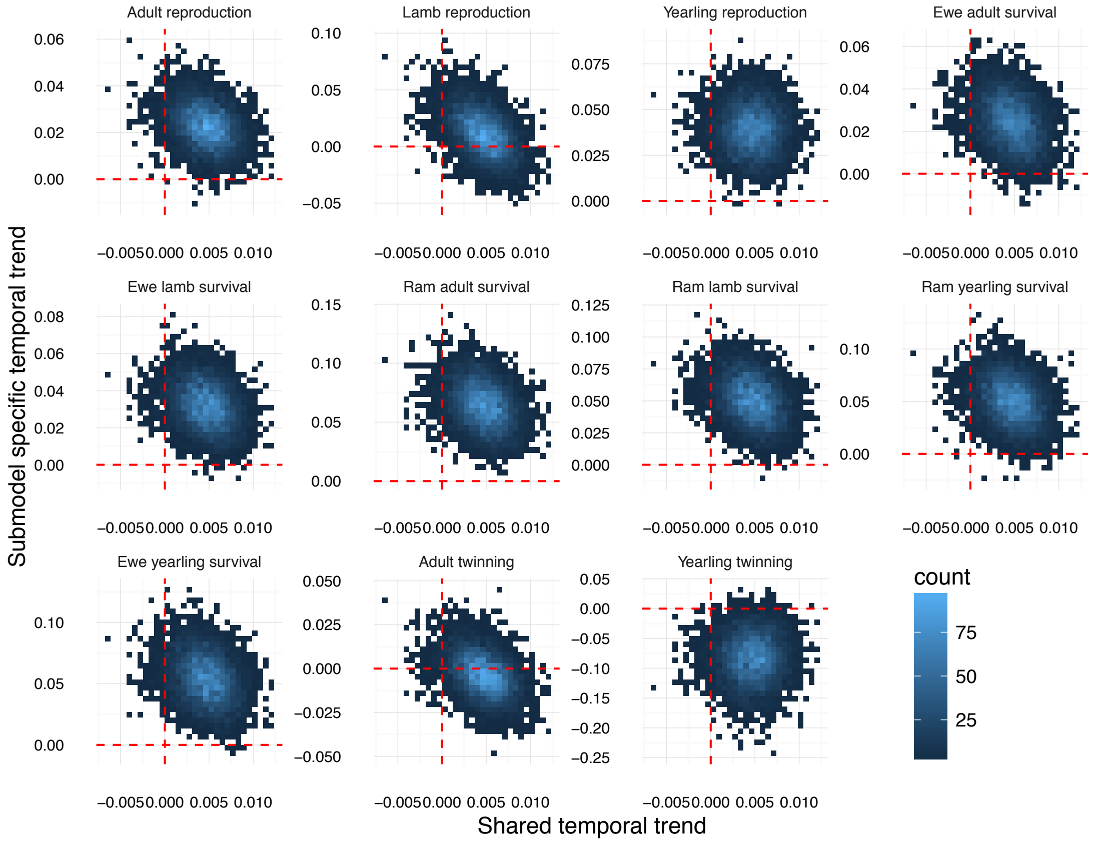

Figure S4: Bivariate plot of the posterior distributions for the two types of temporal trend included in the structural equation model (SEM). Including the shared temporal trend ($\alpha^t t$; see equation 3) in the first environmental axis (e) allows for an interaction between density and time across the vital rates, whilst the submodel specific temporal trends (given by $\beta^t t$; see equations 1-2 & 3) allow for temporal trends in the vital rate means. Both trends were retained as the parameters were not strongly correlated.

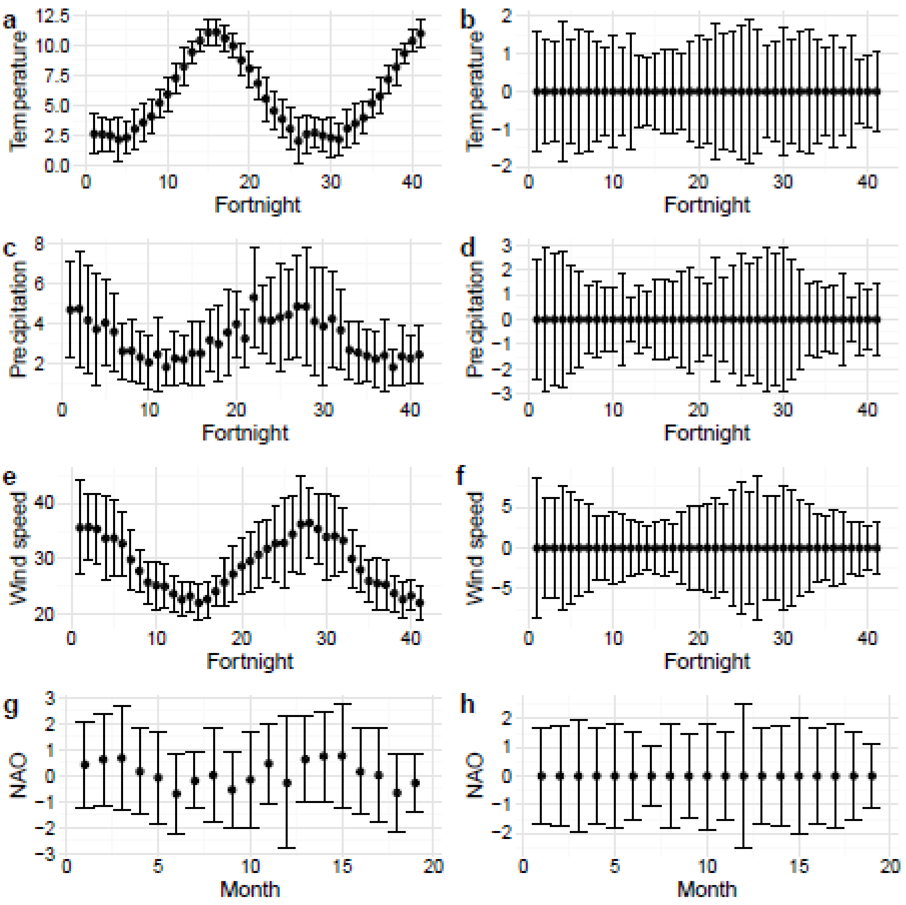

Figure S5: Mean and standard deviation a) temperature (°C), c) precipitation (mm), e) wind speed (knots) and g) NAO. b), d), f) and h) show the mean and standard deviation of the centered climate covariates. The covariates were centered to remove seasonality from the data.

References

Qian, S.S. (2014) Ecological threshold and environmental management: A note on statistical methods for detecting thresholds. *Ecological Indicators,* **38,** 192-197.
