# Supplementary material for: Cumulative weather effects can impact across the whole life cycle
Source: Glob Chang Biol. 2019 Jul 25;25(10):3282–93. doi: 10.1111/gcb.14742 (PMC6771737; doi:10.1111/gcb.14742)
Supplement: Supplementary file 2 [file GCB-25-3282-s002.pdf]

Table S1: Priors for the structural equation models. For the uniform distributions (U) and log-uniform distribution (LU) the first and second parameters refer to the minimum and maximum, for the normal distribution (N) they are the mean and standard deviation. For parameter definitions see eqn 1-4 for parameters in all models and eqn 5-6 for additional parameters in climate models. Note that • is used when referring to parameters for each of the demographic classes, otherwise subscripts indicate which classes are referred to with E for ewes, R for rams, L for lambs, Y for yearlings and A for adults. Weakly informative priors are used to aid convergence of the threshold models, for example by restricting the threshold parameters to the range of observed densities.

Parameter	Model	Submodel(s)	Prior
$\beta_{\bullet}^{0,r}$	All	Reproduction	N(0, 100)
$\beta_{\bullet}^{t,r}$	All	Reproduction	N(0, 1)
$\beta_{\bullet}^{e,r}$	All	Reproduction	N(0, 100)
$\beta_{\bullet}^{f,r}$	All	Lamb and adult reproduction	U(0, 100)
$\beta_{\bullet}^{0,t}$	All	Twinning	N(0, 100)
$\beta_{\bullet}^{t,t}$	All	Twinning	N(0, 1)
$\beta_{\bullet}^{e,t}$	All	Twinning	N(0, 100)
$\beta_{\bullet}^{f,t}$	All	Adult twinning	U(0, 100)
$\beta_{[E]}^{0,s}$	All	Ewe survival	U(-5, 5)
$\beta_{[R]}^{0,s}$	All	Ram yearling and adult survival	U(-10, 10)
$\beta_{[RL]}^{0,s}$	All	Ram lamb survival	N(0, 100)
$\beta_{\bullet}^{t,s}$	All	Survival (except ram lambs)	U(-1, 1)
$\beta_{[RL]}^{t,s}$	All	Ram lamb survival	N(0, 1)
$\beta_{\bullet}^{e,s}$	All	Survival (except ram lambs)	U(0, 50)
$\beta_{[RL]}^{e,s}$	All	Ram lamb survival	N(0,100)
$\theta_{[EL]}$	All	Ewe lamb survival	U(2.2, 2.8)
$\theta_{\bullet}$	All	Survival (except lambs)	U(2.4, 2.8)
σ_e	All	All	U(0, 0.2)
σ_f	All	All	1
ρ_{ef}	All	Adult and lamb reproduction and adult twinning	U(-1, 1)
α^t	All	All	U(-0.05, 0.05)
β^n	Climate	All	U(-0.5, 0.5)
λ	Climate	All	LU(-10, 20)
