# Supplementary material for: Cumulative weather effects can impact across the whole life cycle
Source: Glob Chang Biol. 2019 Jul 25;25(10):3282–93. doi: 10.1111/gcb.14742 (PMC6771737; doi:10.1111/gcb.14742)
Supplement: Supplementary file 3 [file GCB-25-3282-s003.docx]

**Appendix S1: Exploring the dimensionality of the environment**

Posterior predictive checks from the model with a single axis of environmental variation (see main text, equations 1-3) showed that the variation in survival across the six age-sex classes was well explained using a single axis of environmental variation (Fig. 4a). There was some unexplained variation in the fecundity (reproduction and twinning) sub-models (Fig. 4a). Vital rate specific temporal error terms ($\varepsilon_{\bullet,t}^{f}$) were included into the fecundity models with the probability of reproduction now given by

$logit\left( R_{\bullet,t} \right)= \beta_{\bullet}^{0,r}+ \beta_{\bullet}^{t,r}t- \beta_{\bullet}^{e,r}e\left( t \right)+ \varepsilon_{\bullet,t}^{f,r}.$ (eqn A1)

See main text (equations 1-4) for parameter definitions. The twinning models are not shown but were structurally analogous to the reproduction models. The posterior distributions of the $\varepsilon_{\bullet,t}^{f}$ terms suggest that there was residual variation in the lamb reproduction, adult reproduction and adult twinning sub-models (Fig. A1). The posterior distributions for the standard deviations of the error terms in yearling reproduction and twinning are concentrated at zero and these terms were therefore excluded. As the estimates of the vital rate specific error terms were positively correlated the sub-model specific temporal error terms were replaced by a second environmental axis. This accounted for any covariance between lamb reproduction, adult reproduction, and adult twinning not accounted for by the first axis $(e)$. The probability of lamb or adult reproduction therefore becomes

$logit\left( R_{\bullet,t} \right)= \beta_{\bullet}^{0,r}+ \beta_{\bullet}^{t,r}t- \beta_{\bullet}^{e,r}e\left( t \right)+ {\beta_{\bullet}^{f,r}\varepsilon}_{t}^{f},$ (eqn A2)

where$\beta^{f}$ is the slope for the second latent environmental axis ($\varepsilon^{f})$and $\varepsilon^{f}$ and $\varepsilon^{e}$ are sampled from a multivariate normal distribution with means of zero and a covariance matrix $\Sigma= \begin{matrix} \sigma_{e}\sigma_{e} & \sigma_{e}\sigma_{f}\rho_{ef} \\ \sigma_{e}\sigma_{f}\rho_{ef} & \sigma_{f}\sigma_{f} \end{matrix}$. $\sigma_{f}$ was constrained to equal one to make the model identifiable. The structure of the adult twinning model is analogous to the lamb and adult reproduction models. Including the second environmental axis increased the agreement between the observed and predicted fecundity vital rates (Fig. 4b). The first axis of environmental variation affects nine of the vital rates, with the posteriors of the $\beta^{e}$ terms only overlapping zero for yearling reproduction and yearling twinning (Fig. A2). There was no evidence of correlation between the yearly estimates of the second environmental axis and density or the year of study (Fig. A3), suggesting the effects of these variables were accounted for by the first environmental axis $(e)$. There was also no evidence of a correlation with the sex ratio, suggesting the availability of males did not limit female reproduction. This is unsurprising as a single male can fertilise multiple females in any given year (Coltman et al., 1999, Pemberton et al., 1996).

Figure A1: Posterior distributions of the standard deviations of the additional error terms $\varepsilon^{f}$ in the fecundity models (see equation A1).

Figure A2: Posterior estimates (modes and 95% credible intervals) for the slope terms for a) the first and b) the second axes of environmental variation. The superscripts s, r and t refer to whether the parameter is in a survival, reproduction or twinning sub-model. The subscripts give the demographic class, with the first letter referring to the sex (i.e. E for ewe and R for ram) and the second letter the stage class (i.e Lamb, Yearling or Adult).

Figure A3: Correlation between a) the year of the study, b) population size (log 10 number of individuals in August of year *t*), and c) the proportion of rams in the population and the reproductive latent effect ($\varepsilon^{f}$).

**Appendix S2: Incorporating climatic covariates**

*Appendix S2.1 Fitting FLMs*

For the local weather FLMs the means of the daily variables every fortnight (*w*) from the beginning of January in *t*-1 (*w*=1) until the end of July in *t* (*w*=42), were used as covariates (Fig. S5). Monthly NAO data over the same time period were used for the NAO FLM ($w$ = 1, 2…, 19). Seasonality was removed from the weather data by centering (Fig. S5). Each covariate was included in a separate model, with the first environmental axis (*e*) given by:

$e(t)=D_{t}-\sum_{w=1}^{W} f_{c}(w)C_{tw} -\alpha^{t}t- \varepsilon_{t}^{e}$, (eqn 6)

where $C_{tw}$ is climate variable $C$ in year $t$ and time interval $w$ (fortnight for the local variables and month for NAO) and $f_{c}(w)$ is a smooth function that allows the effect of the climate covariates to vary smoothly over the 19 month period (e.g. Fig. 1b). The smooth function is parameterised using spline basis expansion, as $f_{c}\left( w \right)=\sum_{k=1}^{K} \beta_{k}^{c}b_{k}(w)$, where *β^c^* are coefficients, *b*(*w*) are basis functions, and *K* is the dimension of the spline basis. The FLM was estimated using eight knots and a cubic regression (“cr”) spline basis. The degree of smoothing is controlled by a quadratic smoothing penalty, Σ*_j_*λ*_j_β^T^***S***_j_β*, where **S**_j_ are known smoothing penalty matrices and λ_j_ are smoothing parameters (Wood, 2016, Wood, 2017). In a Bayesian framework the FLM coefficients (*β^c^*) can be estimated using a multivariate normal distribution prior, with precision matrix Σ*_j_*λ*_j_***S***_j_* (Wood, 2016, Wood, 2017). The smoothing parameters, λ_j_, were estimated as parameters in the model using vague log-uniform priors (Table S1). The *jagam* function in the *mgcv* package (Wood, 2016) was used to generate the smoothing penalty matrices (**S**) and the spline bases (*b*(*w*)).

*S2.2 Climatic effects in the reproductive latent effect*

As the first axis of environmental variation affected both survival and fecundity (Fig. A3) we assumed that this accounted for temporal variation in reproduction due to the selective mortality of reproductive individuals. That is we assume that for example a lamb that is pregnant has a higher mortality risk in a ‘bad’ winter than a lamb that is not pregnant and therefore the proportion of lambs reproducing is lower in ‘bad’ winters as a higher proportion of pregnant lambs have died. As the second environmental axis of temporal variation only affects reproduction (Fig. A3) we assume that this affects the probability of individuals conceiving, for example by affecting the condition of the sheep entering the rutting period. Climatic covariates were therefore included from January $t-1$ until November in year $t$, when the rut occurs. The same local variables were included as in the first environmental axis (minimum temperature, precipitation and maximum wind speed).

The probability of lamb reproduction or adult reproduction is now given by

$logit\left( R_{\bullet,t} \right)= \beta_{\bullet}^{0,r}+ \beta_{\bullet}^{t,r}t- \beta_{\bullet}^{e,r}e\left( t \right)+ \beta_{\bullet}^{f,r}f(t),$ (eqn A3)

where $f(t)=\sum_{k=1}^{K} \beta_{k}^{c}b_{k}\left( C_{t} \right)+ \varepsilon_{t}^{f}$ and $\beta_{\bullet}^{f,r}$ is a slope parameter. The remaining parameters are defined in equation 6 and details on fitting the model are the same as those provided in the main text. The adult twinning model is structurally the same as equation A3 so is not shown. Six knots were used for the spline. Weather variables were not included in the first axis of environmental variation (i.e. $e$ is given by equation 3 in the main text).

Precipitation, wind speed and monthly NAO did not appear to act on the second axis of environmental variation (Fig. A4). There was some evidence that increased temperatures over the spring and summer preceding the rut may increase fecundity (Fig. A4a). Higher temperatures over this period may increase vegetation growth, therefore increasing resource availability and the condition in which the ewes enter the rut. However cross validation (see equation 7) showed that including the temperature FLM did not improve the predictive performance of the base model ($\hat{elpd}= -864.4$ for temperature model and $\hat{elpd}= -863.9$ for base model).

****Figure A4: Functional linear models for the second axis of environmental variation with a) temperature, b) precipitation, c) wind speed and d) NAO. Thick black lines show the posterior medians, thinner grey lines show 100 simulations from the posterior. The horizontal dashed red line is at 0. Dashed vertical lines and letters at the top of the plot indicate the seasons. Coefficients above the line indicate that higher values of the climatic covariate during that time period were associated with an increase in fecundity.

*S2.3 Incorporating multiple local climatic covariates in the first axis of environmental variation*

The predictive performance of both the wind speed and precipitation FLMs was better than the baseline model (Table 1). As such we modelled the first temporal axis of variation $(e)$ as a function of both wind speed $(W)$and precipitation $(P)$ as follows

$e(t)=D_{t}-\sum_{k=1}^{K} \beta_{k}^{w}b_{k}(W_{t})-\sum_{k=1}^{K} \beta_{k}^{p}b_{k}(P_{t}) -\alpha^{t}t- \varepsilon_{t}^{e}.$ (eqn A4)

where $D_{t}$ is the density in year $t$, $\alpha^{t}$is a temporal trend, and $\beta^{w}$and $\beta^{p}$ are coefficients, estimated using spline basis expansion as in the main text (equation 6). $\varepsilon_{t}^{e}$ accounts for any residual temporal covariation in the vital rates. Cross validation was performed as in the main text (equation 7) and the predictive performance of this model was compared to the single climatic covariate wind speed and precipitation models.

Similar effects are seen for each climatic covariate when wind speed and precipitation are included in the same model as when these are modelled separately (Fig. A5). Higher precipitation generally has a negative effect on the vital rates whilst higher wind speeds have a positive effect in Spring year $t-1$and negative over autumn and winter in year $t$. However, the effect sizes are decreased for both climatic covariates in the joint model, relative to including each climatic covariate in a separate model (Fig. A5). This is unsurprising as the wind speed and precipitation are correlated (r=0.71, p<0.01). Climatic covariates are often correlated with each other (Grosbois et al., 2008), sometimes making it difficult to determine variables are drivers and which are simply correlated with drivers (Ehrlen et al., 2016). Including both variables does not improve the predictive ability of the model beyond that of the wind speed only model (Table A1). Here an additive relationship between the climatic covariates was assumed. In reality it is often an interaction between climatic covariates that affects the vital rates (Stenseth and Mysterud, 2005), for example here wet and windy weather may be a lot worse thermodynamically than the additive effect would suggest.

**

Figure A5: FLM with two climatic covariates; a) wind speed and b) precipitation (see equation A4). Thick black lines show the posterior medians, thinner grey lines show 100 simulations from the posterior. Thin dashed black lines show the medians from the respective single climatic variable FLMs. The horizontal dashed red line is at 0. Dashed vertical lines and letters at the top of the plot indicate the seasons. Coefficients above the line indicate that higher values of the climatic covariate during that time period were associated with an increase in fecundity.

Table A1: Difference in the predictive ability of the model with the highest predictive ability (wind speed) and the other models on a deviance scale. Lower values indicate models with a higher predictive ability.

| **Model** | **Relative predictive ability** | **R^2^** |
| --- | --- | --- |
| Precipitation | 1.91 | 0.77 (0.65-0.84) |
| Precipitation and wind speed | 1.56 | 0.81 (0.70-0.88) |
| Wind speed | 0.00 | 0.81 (0.69-0.87) |

*S2.4 Incorporating broadscale and local climatic covariates*

Including winter NAO and the local weather variables in a single model can determine whether the FLMs are able to identify effects beyond those seen in the winter NAO model. Here the first axis of environmental variation is given by

$e(t)=D_{t}-\beta^{N}{NAO}_{t}-\sum_{k=1}^{K} \beta_{k}^{c}b_{k}(C_{t}) -\alpha^{t}t- \varepsilon_{t}^{e},$ (eqn A5)

where ${NAO}_{t}$ is winter NAO in year $t$, $\beta^{N}$ is a slope term and the remaining parameters are defined in equation 6 and details on fitting the model are the same as those provided in the main text. This was repeated using both precipitation and wind speed as the local variable $(C)$.

Including the winter NAO term decreases the magnitude of the coefficients for both precipitation and wind speed (Fig. A6). The largest decreases are during winter, as would be expected given that high winter NAO values are associated with wet and windy winters. However, it is not only the magnitude of the coefficients during winter that is altered, possibly because weather may be correlated within years and changes in the winter NAO term may also therefore be correlated with changes in the weather outside of winter. Including the local weather variables does not change the magnitude of the $\beta^{N}$ slope term in either model (Fig. A6). The models including the local covariates in addition to the winter NAO term have a higher predictive ability than the model with just the winter NAO term, however this is marginal, especially in the case of the precipitation model (Table A2).

Figure A6: Estimates of climatic coefficients for the wind speed and winter NAO model (top row) and the precipitation and winter NAO model (bottom row). a) and c) show the FLMs for windspeed and precipitation respectively. Thick black lines show the posterior medians, thinner grey lines show 100 simulations from the posterior. Thin dashed black lines show the medians from the respective single weather variable FLMs. The horizontal dashed red line is at 0. Dashed vertical lines and letters at the top of the plot indicate the seasons. Coefficients above the line indicate that higher values of the weather variable during that time period were associated with an increase in survival and fecundity. b) and d) show the posterior distribution for $\beta^{N}$. The vertical red line shows the equivalent parameter from the model with winter NAO only (i.e. without a local covariate).

Table A2: Difference in the predictive ability of model with the highest predictive ability (wind speed and winter NAO) and the other models on a deviance scale. Lower values indicate models with a higher predictive ability. R^2^ is the proportion of variation in the first environmental axis (*e*) explained by the fixed effects (i.e. density, the temporal trend, and the relevant climatic variables). Values are the median and 95% quantiles, calculated by sampling from the posterior distribution. R^2^ for the base model is 0.68 (0.57-0.74).

| **Model** | **Relative predictive ability** | **R^2^** |
| --- | --- | --- |
| Precipitation | 11.00 | 0.77 (0.65-0.84) |
| Wind speed | 9.09 | 0.81 (0.69-0.87) |
| Winter NAO | 1.24 | 0.86 (0.79-0.90) |
| Precipitation + Winter NAO | 1.17 | 0.86 (0.79-0.91) |
| Wind speed + Winter NAO | 0.00 | 0.87 (0.80-0.91) |

**References**

Coltman, D. W., Bancroft, D. R., Robertson, A., Smith, J. A., Clutton-Brock, T. H. and Pemberton, J. M. (1999) 'Male reproductive success in a promiscuous mammal: behavioural estimates compared with genetic paternity', *Molecular Ecology,* 8(7), pp. 1199-1209.

Ehrlen, J., Morris, W. F., von Euler, T. and Dahlgren, J. P. (2016) 'Advancing environmentally explicit structured population models of plants', *Journal of Ecology,* 104(2), pp. 292-305.

Grosbois, V., Gimenez, O., Gaillard, J. M., Pradel, R., Barbraud, C., Clobert, J., Moller, A. P. and Weimerskirch, H. (2008) 'Assessing the impact of climate variation on survival in vertebrate populations', *Biological Reviews,* 83(3), pp. 357-399.

Pemberton, J. M., Smith, J. A., Coulson, T. N., Marshall, T. C., Slate, J., Paterson, S., Albon, S. D. and CluttonBrock, T. H. (1996) 'The maintenance of genetic polymorphism in small island populations: Large mammals in the Hebrides', *Philosophical Transactions of the Royal Society of London Series B-Biological Sciences,* 351(1341), pp. 745-752.

Stenseth, N. C. and Mysterud, A. (2005) 'Weather packages: finding the right scale and composition of climate in ecology', *Journal of Animal Ecology,* 74(6), pp. 1195-1198.

Wood, S. N. (2016) 'Just Another Gibbs Additive Modeler: Interfacing JAGS and mgcv', *Journal of Statistical Software,* 75(7), pp. 1-15.

Wood, S. N. 2017. Generalized Additive Models: An Introduction with R. 2nd Edition ed.: Chapman and Hall/CRC.
